# HASPIN kinase inhibitor CHR-6494 suppresses intestinal polyp development, cachexia, and hypogonadism in *Apc*^*min/+*^ mice

**DOI:** 10.1097/CEJ.0000000000000562

**Published:** 2019-12-17

**Authors:** Hiromitsu Tanaka, Morimasa Wada, Junhyeok Park

**Affiliations:** Faculty of Pharmaceutical Sciences, Nagasaki International University, Sasebo, Nagasaki, Japan

**Keywords:** cancer, chemoprevention, infertility, testis, testosterone

## Abstract

HASPIN has been identified as a nuclear Ser/Thr kinase specifically expressed in haploid germ cells. HASPIN kinase inhibitors were recently isolated, and their antitumor activity reported. Colorectal cancer occurs with high incidence worldwide. In this study, we examined whether HASPIN inhibitor CHR-6494 suppresses cancer progression in *Apc*^*Min/+*^ mice, a familial colon tumor disease model. Mice were treated by intraperitoneal injection of CHR-6494 for 50 days. Following the treatment period, intestinal polyps were counted and testosterone and spermatogenesis levels were observed. Intraperitoneal administration of CHR-6494 significantly inhibited intestinal polyp development and recovered body weight in *Apc*^*Min/+*^ mice. Although spermatogenesis was inhibited with increasing age in *Apc*^*Min*/+^ mice, CHR-6494 significantly improved blood testosterone levels and spermatogenesis. Our results suggest that HASPIN inhibitors may be useful as anti-cancer agents and for the treatment of hypogonadism in colorectal cancer patients.

## Introduction

Mouse *haspin* (known as Gsg2) has been cloned as a gene encoding the nuclear Ser/Thr kinase-subtracted cDNA library, derived from W/W^v^ mutant testis cells of wild-type (WT) testis cDNAs (Tanaka *et al.*, 1994; Tanaka *et al.*, 1999). HASPIN has been suggested to phosphorylate histone H3 at threonine 3 in mitotic cells (Dai *et al.*, 2005; Dai and Higgins, 2005). HASPIN is found in the centrosomes and spindles during mitosis, where it integrates the regulation of chromosome and spindle function during mitosis and meiosis (Yu *et al.*, 2017; Cao *et al.*, 2019). Recently, HASPIN was detected during phosphorylation of threonine 127 on TH2A, a germ cell-specific H2A variant, in condensed spermatids and mitotic early preimplantation mouse embryos (Hada *et al.*, 2017). To clarify the role of Haspin, *haspin* gene-disrupted mice were generated, but no distinct phenotype was observed (Shimada *et al.*, 2016). Microscopic observation of the motility of *haspin*-disrupted sperm showed that the sperm were not abnormal, although seminiferous tubules lacking germ cells were occasionally observed (Shimada *et al.*, 2016). Inhibitors of HASPIN kinase activity have also been reported to suppress cancer growth (Huertas *et al.*, 2012; Kim *et al.*, 2017; Opoku-Temeng *et al.*, 2018). Experiments using cultured cells and *haspin*-disrupted mice have indicated that HASPIN functions may be compensated by other molecules in normal cells; however, HASPIN may play an important role in cell division during the proliferation of male germ cells and cancer cells.

Human colorectal cancer (CRC) is a major cause of death worldwide (Jackstadt and Sansom, 2016); although treatments such as polyp excision have been established, the development of a more effective treatment is required. *Apc* is a tumor-suppressing *adenomatous polyposis coli* gene that is involved in the earliest stage of CRC development. *Apc*^*Min/+*^ mice are a familial colon cancer mouse model with a nonsense mutation at codon 850 in the *Apc* gene (Tomita *et al.*, 2007). APC protein forms a complex with various other proteins and is involved in the control of signal transduction pathways (Tomita *et al.*, 2007; Jackstadt and Sansom, 2016). *APC* may be a tumor-suppressing gene involved in the earliest stage of CRC development. In a previous study, approximately 25–75 adenomas (polyps) were found to develop in the small intestine in *Apc*^*Min/+*^ mice at 160–180 days of age, with cachexia and hypogonadism, gradually progressing during tumor development (White *et al.*, 2013).

In this study, we observed polyp formation in *Apc*^*Min/+*^ mice following administration of HASPIN inhibitor CHR-6494 to determine whether it can prevent cancer growth *in vivo*.

## Methods

### Animals

*Apc*^*Min/+*^ (C57BL/6J) mice were developed by Jackson Laboratories (Bar Harbor, Maine, USA). In this study, C57BL/6J mice were purchased from Japan SLC (Shizuoka, Japan) and sacrificed by cervical dislocation immediately before experiments. All animal experiments conformed to the Guide for the Care and Use of Laboratory Animals and were approved by the Institutional Committee of Laboratory Animal Experimentation and Research Ethics Committee of Nagasaki International University. This article does not contain any experiments with human subjects performed by any of the authors. Mice were maintained under specific pathogen-free conditions in the animal experimentation facility at Nagasaki International University, with temperature and lighting controlled throughout the experimental period. Mice were provided with food and water *ad libitum*.

### Administration of CHR-6494

**Fig. 1:**
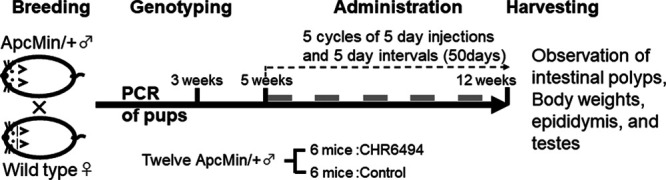
Schematic representation of the experimental procedure. Treatments were performed for five cycles consisting of five consecutive injection days (gray bars) and five consecutive naive days over a period of 50 days.

The experimental procedure is schematically shown in Fig. 1. *Apc*^*Min/+*^ males were bred with WT females, and the tails of the pups were subjected to PCR by 3 weeks of age under the conditions described in a previous study (Mochida *et al.*, 2003). The *Apc*^*Min/+*^ male pups were selected. Experiments were performed on 12 *Apc*^*Min/+*^ males, randomized into a control group and a treatment group. In the control group, six mice were treated with a 200-µl intraperitoneal injection of a vehicle solution of 10% dimethyl sulfoxide (DMSO) and 20% 2-hydroxypropyl-b-cyclodextrin (Sigma-Aldrich, Tokyo, Japan) beginning at the age of 5 weeks. In the treatment group, six mice were treated with a 200-µl intraperitoneal injection of 50 mg/kg CHR-6494 (Cayman Chemical, Ann Arbor, Michigan, USA) diluted with a final concentration of 10% DMSO and 20% 2-hydroxypropyl-b-cyclodextrin beginning at the age of 5 weeks. The CHR-6494 (5 µg/µl) was stored in 100% DMSO at −30°C. The injectable material included 200 µl of fresh 100% 2-hydroxypropyl-b-cyclodextrin and 100 µl of 5-µg/µl CHR-6494 adjusted to 1 ml with saline. The injection volume was adjusted according to the weight of each mouse. Treatments were performed for five cycles consisting of five consecutive injection days and five consecutive naive days over a period of 50 days. The treatments followed previous drug treatment procedures (Mochida *et al.*, 2003; Huertas *et al.*, 2012).

### Counts of intestinal polyps

Intestinal polyps were counted according to previously published methods (Mochida *et al.*, 2003). Freshly removed small intestines were each divided into five equal segments. These segments were incised longitudinally, washed with PBS, laid flat on filter paper, and fixed for 24 hours in 10% neutral-buffered formalin. Fixed intestinal segments were stained with 1% methylene blue and examined for tumors by gross inspection and light microscopy.

### Histological observation of testes and epididymides

Bouin’s solution-fixed mouse testes were cut into 7-μm-thick sections and mounted on silane-coated slides. Slides were then stained and examined under a microscope.

### Serum testosterone measurements

Serum testosterone levels were measured by liquid chromatography-tandem mass spectrometry (LC-MS/MS) methods (Oriental Yeast Co., Ltd., Shiga, Japan).

### Statistical analyses

Data are expressed as means ± SD. Means were compared between treatment groups using Student’s *t*-test; significant differences were determined at the level of *P* < 0.05.

## Results

### Intestinal polyps

**Fig. 2:**
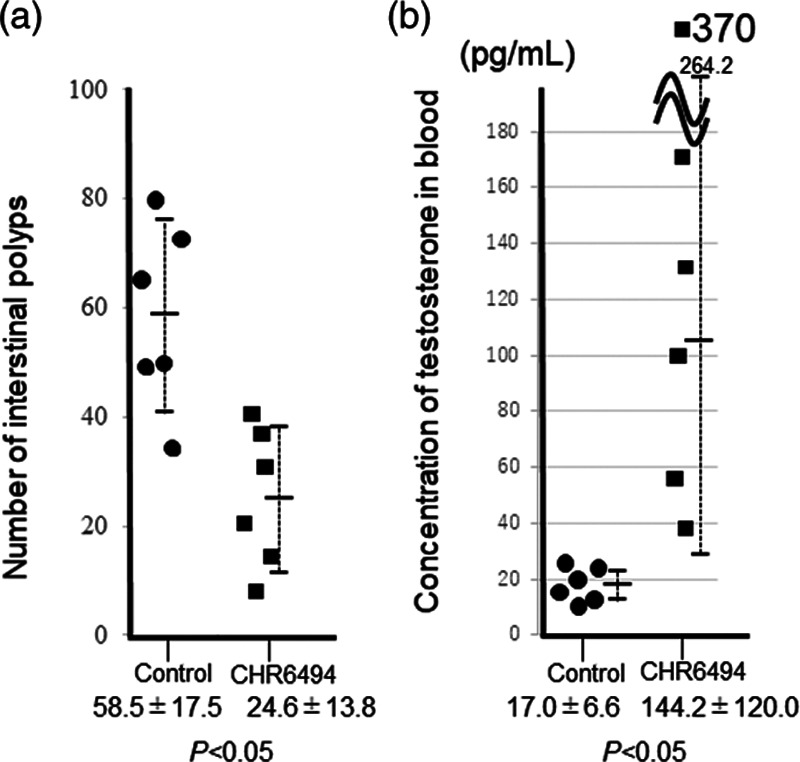
Numbers of intestinal polyps in *Apc*^*min/+*^ mice treated with CHR-6496 (a) and Testosterone levels in blood from *Apc*^*min/+*^ mice treated with CHR-6496 (b). Dots and squares indicate results in treated and untreated mice, respectively. Testosterone levels were examined using liquid chromatography-tandem mass spectrometry (LC-MS/MS). In control mice, the mean testosterone level was 221.0 ± 47.1 pg/ml (Steinfeld, 2018).

The numbers of intestinal polyps observed in 5-week-old *Apc*^*Min/+*^ mice treated with and without CHR-6494 for 7 weeks are shown in Fig. 2a. Significantly fewer polyps were observed in the small intestines of mice treated with CHR-6494 (24.6 ± 13.8) than in those of control mice (58.5 ± 17.5). No special polyp, body weight, or testis phenotypes were observed in C57BL/6J WT mice treated with CHR-6494.

### Body weights

*Apc*^*Min/+*^ mice developed cachexia during the treatment period (White *et al.*, 2013). We measured body weight in 12-week-old mice at the end of the experiment. Body-weight was significantly recovered in mice treated with CHR-6494, at 23.6 ± 4.2 g, compared with 18.8 ± 1.7 g in control mice (Table 1).

**Table 1:** Effect of CHR6496 treatment on body and testicular weights

	Wild type	*Apc*^*Min/+*^	CHR6494^a^
Body weight (g)	26.4 ± 1.5	18.8 ± 1.7	23.6 ± 4.2 (0.03)
Testicular weight (g)	0.09 ± 0.01	0.04 ± 0.03	0.08 ± 0.03 (0.006)
Epididymal weight (g)	0.012 ± 0.0007	0.006 ± 0.0014	0.0097 ± 0.0012 (2.1E-06)

The numbers of in parentheses indicated p values in *Apc*^*Min/+*^ and CHR6494.

a CHR6494 mean *Apc*^*Min/+ min*^ mice treated with CHR6494.

### Observation of testes and epididymides

**Fig. 3:**
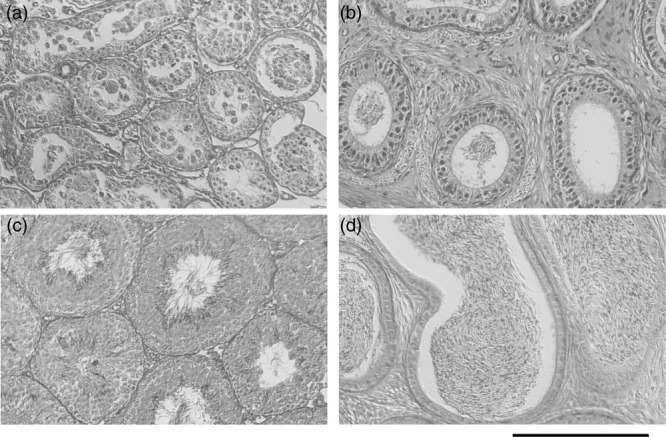
Sections of Bouin’s solution-fixed testes (a and c) and epididymides stained (b and d) with hematoxylin and eosin. Sperm were not included in testes (a) and epididymides (b) of *Apc*^*min/+*^ mice. Spermatogenesis was fully recovered in the testes (a) and epididymides (b) of *Apc*^*min/+*^ mice treated with CHR-6496. Bar = 200 μm.

*Apc*^*Min/+*^ mice developed hypogonadism (Fig. 3a and b) (You *et al.*, 2006; White *et al.*, 2013). Testicular and epididymal weights were significantly recovered in mice treated with CHR-6494, at 0.08 ± 0.03 and 0.0097 ± 0.0012 g, respectively, compared with 0.04 ± 0.03 and 0.006 ± 0.0014 g, respectively, in control mice (Table 1). Histochemical observation showed that spermatogenesis was recovered from hypogonadism in *Apc*^*Min/+*^ mice treated with CHR-6494 (Fig. 3c and d). Testosterone levels were significantly recovered in 12-week-old mice treated with CHR-6494, at 142.6 ± 118.3 pg/ml, compared with 17.0 ± 6.6 pg/ml in control mice (Fig. 2b).

## Discussion

HASPIN has been identified as a nuclear serine-threonine kinase, and has been cloned in humans (Tanaka *et al.*, 1999; Tanaka *et al.*, 2001). HASPIN is expressed in trace amounts in various organs (Higgins, 2001a), and the function and evolution of *haspin* genes among different organisms is the subject of considerable research interest (Higgins, 2001b). Experiments with cultured cells have shown that phosphorylated histone H3 functions in chromosome distribution (Yu *et al.*, 2017), whereas histone H2A is phosphorylated in germ cells (Hada *et al.*, 2017). The generation of *haspin*-disrupted mice has demonstrated that germ cells and organisms are maintained even without *haspin* (Shimada *et al.*, 2016). Seminiferous tubules with abnormal germ cells, which are not found in WT, are often found in *haspin*-disrupted mice (Shimada *et al.*, 2016). Together, the findings of these previous studies suggest that HASPIN’s function is compensated by other molecules during normal cell differentiation and proliferation. Recently, HASPIN inhibitors have been shown to inhibit cancer cell growth (Huertas *et al.*, 2012), suggesting that HASPIN’s function is not complemented during cell division in actively proliferating cells.

To investigate whether HASPIN inhibitor CHR-6494 can suppress cancer cell growth *in vivo*, we administered CHR-6494 to *Apc*^*Min/+*^ mice, a model of human CRC. *Apc*^*min/+*^ mice developed intestinal polyps, cachexia, and hypogonadism with age (You *et al.*, 2006; White *et al.*, 2013; Jackstadt and Sansom, 2016). Our results indicate that CHR-6494 administration significantly suppressed intestinal polyp development, cachexia, and hypogonadism in *Apc*^*min/+*^ mice. Intestinal polyp development involved extensive cell proliferation. Thus, HASPIN’s function, which is required for cell growth, was not complemented by other molecules in *Apc*^*min/+*^ mice treated with CHR-6494, and intestinal polyp development was suppressed.

Although the cause of germ cell abnormality in *Apc*^*min/+*^ mice is unknown, Leydig cells in *Apc*^*min/+*^ mice are abnormal in that they do not develop tumors due to a decrease in testosterone. APC is involved in the Wnt signal (Tomita *et al.*, 2007); testicular cells including Leydig cells are maintained by the Wnt/β-catenin signal (Qian *et al.*, 2013). Disruption of the Wnt signaling network may disarrange testicular cells, although it remains unclear whether this effect is direct or indirect. That is, HASPIN may directly disturb signal transduction abnormalities in *Apc*^*min/+*^ mouse testicular cells through avoidance behavior. Alternatively, testicular cells may cause dysfunction via abnormal cytoadhesin or humoral factors in *Apc*^*min/+*^ mice.

In conclusion, although our results arise from a small sample, they indicate that HASPIN inhibitors may be useful as anti-cancer agents and for the treatment of hypogonadism in CRC. The significance of the results of the present study requires further validations in humans.

## Acknowledgements

**Conflicts of interest**

There are no conflicts of interest.
